# Supplementary material for: Carbon source, cell density, and the microbial community control inhibition of V. cholerae surface colonization by environmental nitrate
Source: mBio. 2025 Feb 25;16(4):e04066-24. doi: 10.1128/mbio.04066-24 (PMC11980369; doi:10.1128/mbio.04066-24)
Supplement: Supplemental figures — Figure S1–S6. [file mbio.04066-24-s0001.pdf]

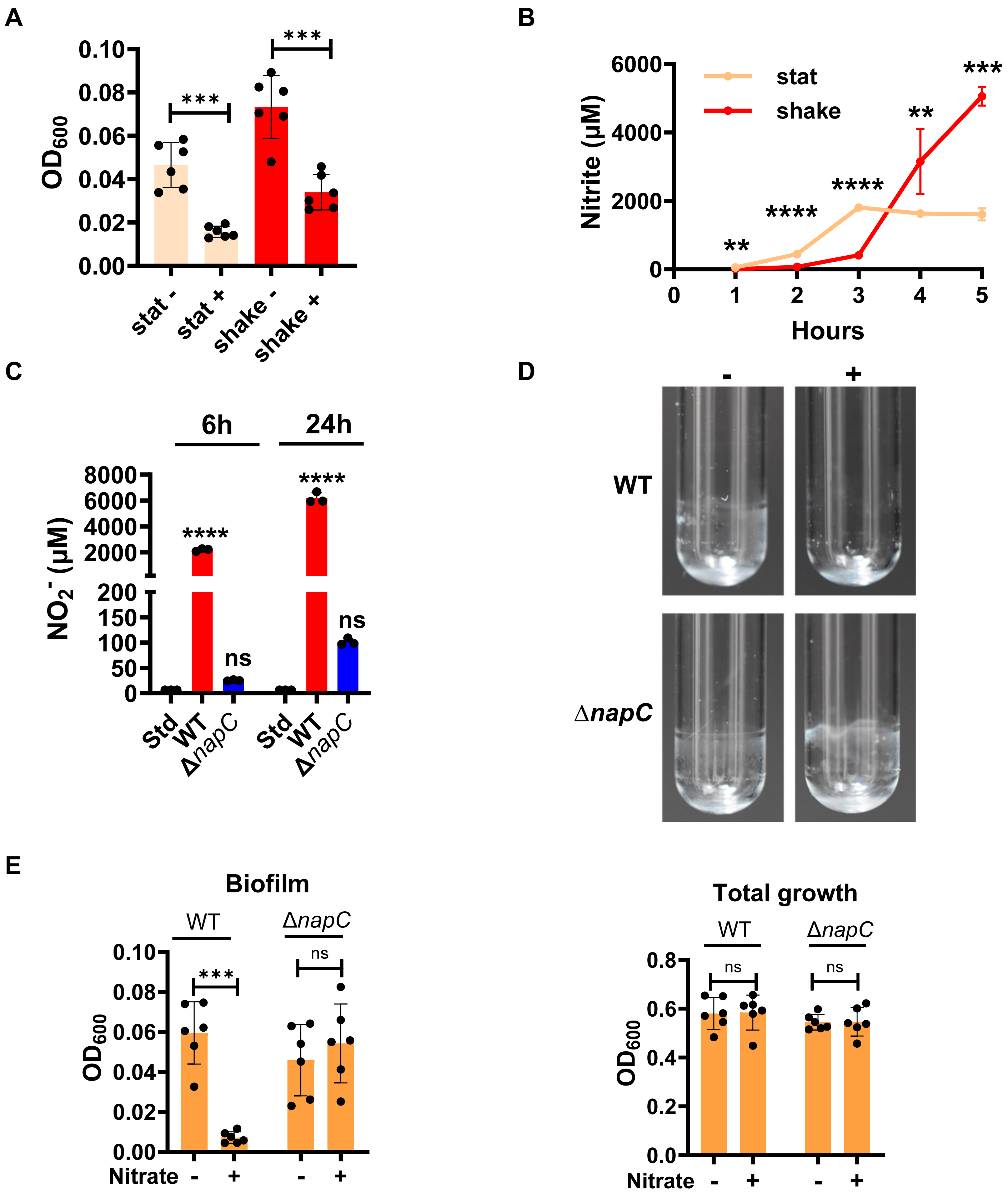
A
OD600
0.10
0.08
0.06
0.04
0.02
0.00
***
***
stat -
stat +
shake -
shake +
B
stat
shake
6000
4000
2000
0
Nitrite (μM)
**
****
****
**
***
0
1
2
3
4
5
Hours
C
6h
24h
8000
6000
4000
2000
200
150
100
50
0
NO2- (μM)
****
****
ns
ns
Std
WT
ΔnapC
Std
WT
ΔnapC
D
-
+
WT
ΔnapC
E
Biofilm
WT
ΔnapC
0.10
0.08
0.06
0.04
0.02
0.00
OD600
***
ns
Nitrate
-
+
-
+
Total growth
WT
ΔnapC
0.8
0.6
0.4
0.2
0.0
OD600
ns
ns
Nitrate
-
+
-
+

Figure S1: Nitrate reduction to nitrite is essential for inhibition of biofilm accumulation by nitrate. (A) An expanded view of the WT *V. cholerae* biofilm measurement shown in Fig 1D. (B) Nitrite generation by WT *V. cholerae* under static and aerated conditions. The mean of biological triplicates is shown. Error bars represent the standard deviation. Significance at each time point was evaluated using a Welch's t test. (C) Quantification of nitrite (NO_2^-) generation in LB supplemented with nitrate by WT *V. cholerae* and a Δ*napC* mutant after 6 and 24 hours of incubation. Std indicates a 5 mM nitrate in LB standard without bacteria added. The mean of biological triplicates is shown. Error bars represent the standard deviation. An ordinary one-way ANOVA with Dunnett's test was used to determine whether measurements differed significantly from the standard. (D) Images of biofilms formed by WT *V. cholerae* and a Δ*napC* mutant in LB alone (-) or supplemented with 5 mM nitrate (+). (E) Quantification of biofilm formation and total growth by the strains in (D). The mean of six biological replicates is shown. Error bars represent the standard deviation. Significance was calculated using a Welch's t test for biofilm accumulation and a student's t test for total growth. ****$p<0.0001$, ***$p<0.001$, ** $p<0.01$, ns not significant.

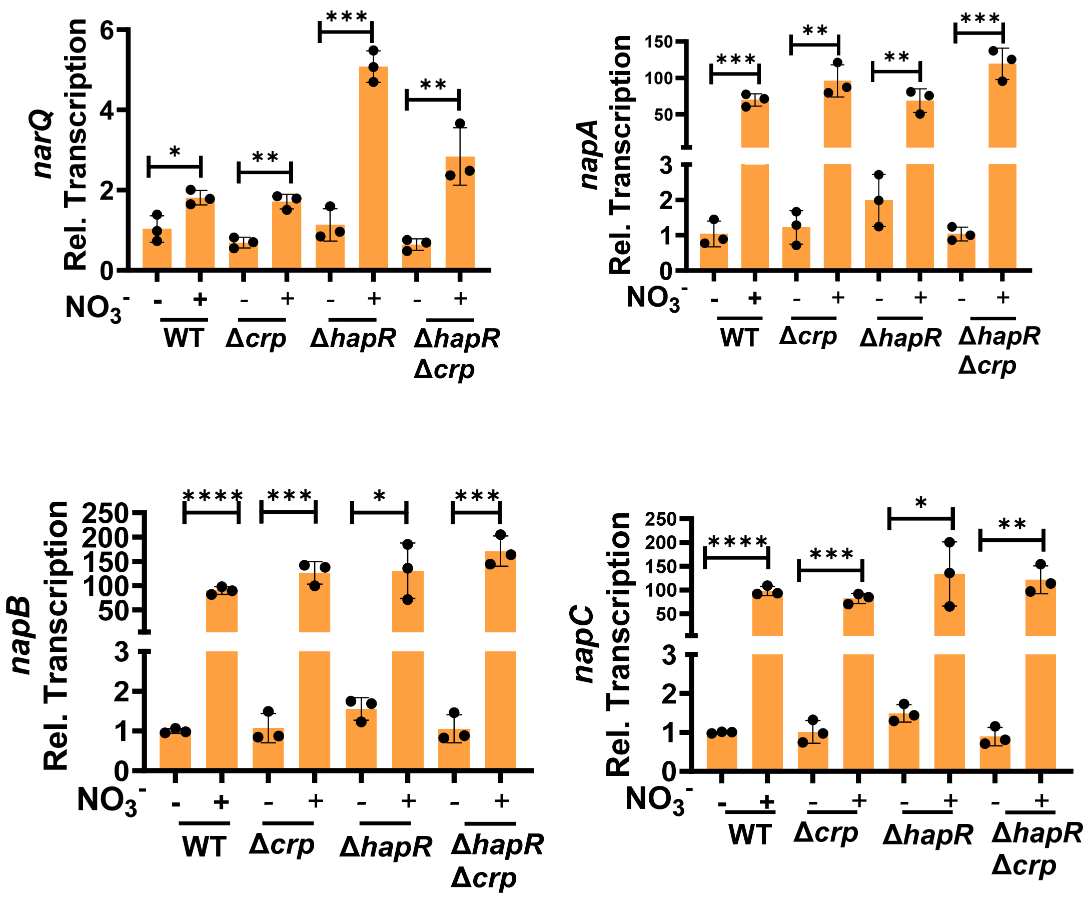

Figure S2: Expression of the *narQ* and the *nap* genes increases in response to nitrate supplementation in static cultures of WT *V. cholerae* and mutants. qRT-PCR quantification of transcription of the *V. cholerae* genes required for nitrite generation in LB alone (-) and supplemented with 5 mM nitrate (+). The indicated strains were cultured statically and harvested after 8 hours. The mean of biological triplicates is shown for all experiments. Error bars reflect the standard deviation. Significance was calculated using a student's t test. **** p<0.0001, *** p<0.001, ** p<0.01, * p<0.05, ns not significant.

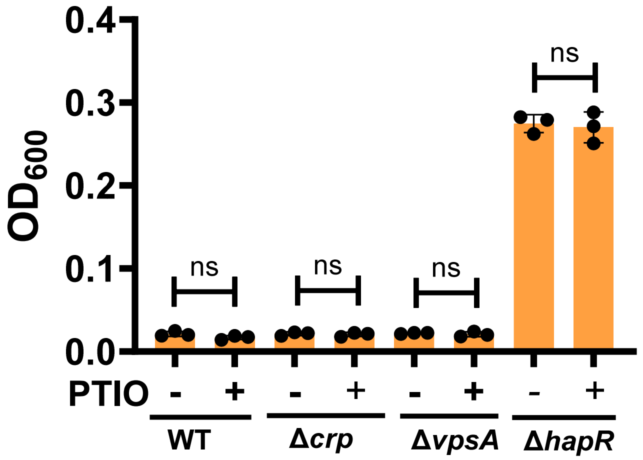

Figure S3: The nitric oxide scavenger PTIO does not rescue inhibition of *V. cholerae* biofilm formation by nitrite. Quantification of biofilms formed by WT *V. cholerae* and the indicated mutants cultured statically in LB supplemented with 5 mM nitrite alone (-) or with addition of the nitric oxide scavenger 2-phenyl-4,4,5,5-tetramethylimidazoline-1-oxyl-3-oxide (PTIO, 500 μM) (+). The mean of biological triplicates is shown. Error bars reflect the standard deviation. Significance was calculated using a student's t test. ns not significant.

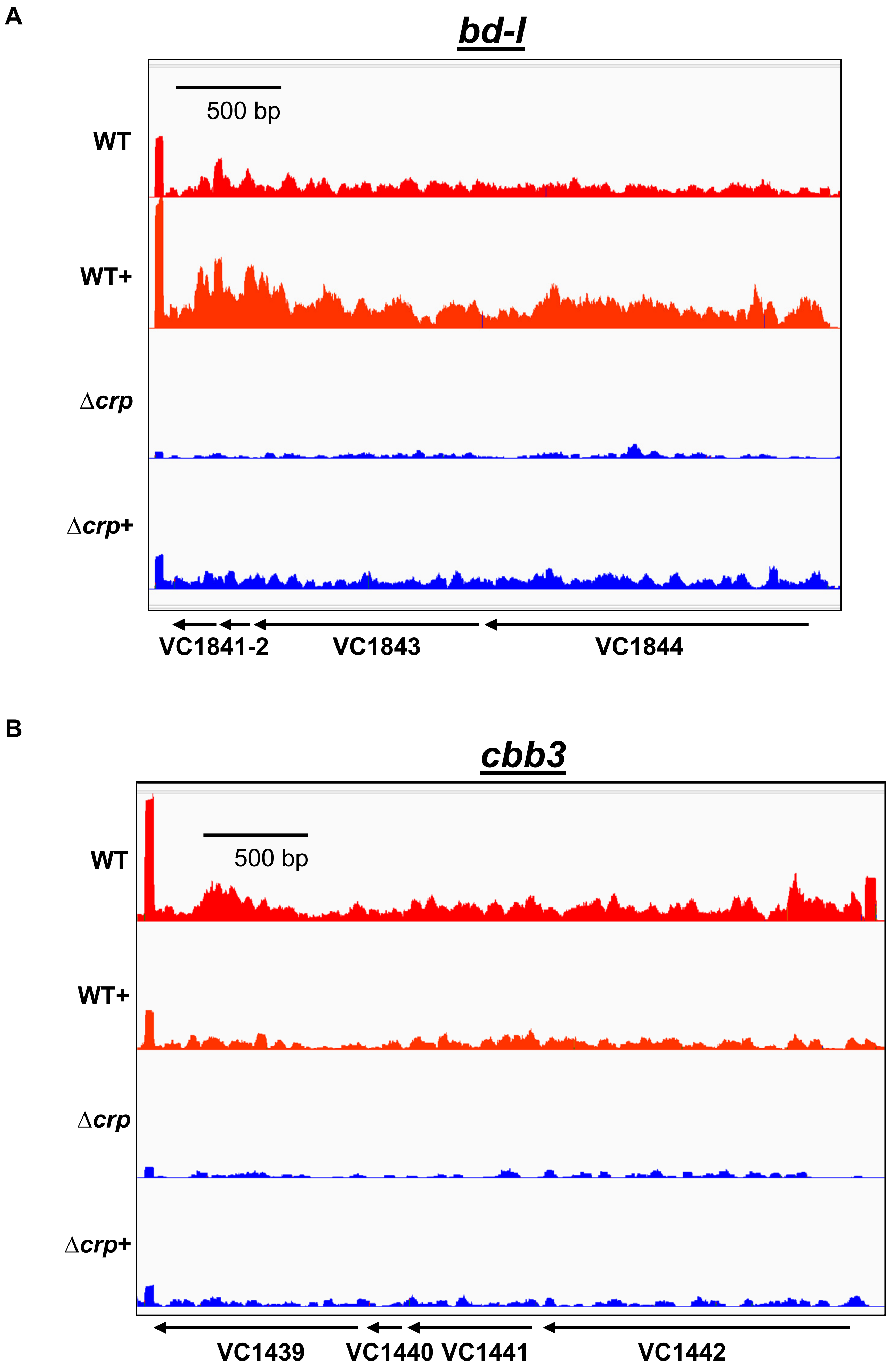
A
bd-I
500 bp
WT
WT+
Δcrp
Δcrp+
VC1841-2
VC1843
VC1844
B
cbb3
500 bp
WT
WT+
Δcrp
Δcrp+
VC1439
VC1440
VC1441
VC1442

Figure S4: CRP activates transcription of the principal aerobic terminal oxidases of *V. cholerae*. Normalized RNAseq data showing transcription of the principal aerobic terminal oxidases of *V. cholerae* (A) *bd-I* and (B) *cbb3* in WT *V. cholerae* and the Δ*crp* mutant. Cells were cultured statically in Lb broth alone (-) or supplemented with 5 mM nitrate (+). Experiments were performed in triplicate. A representative trace is shown.

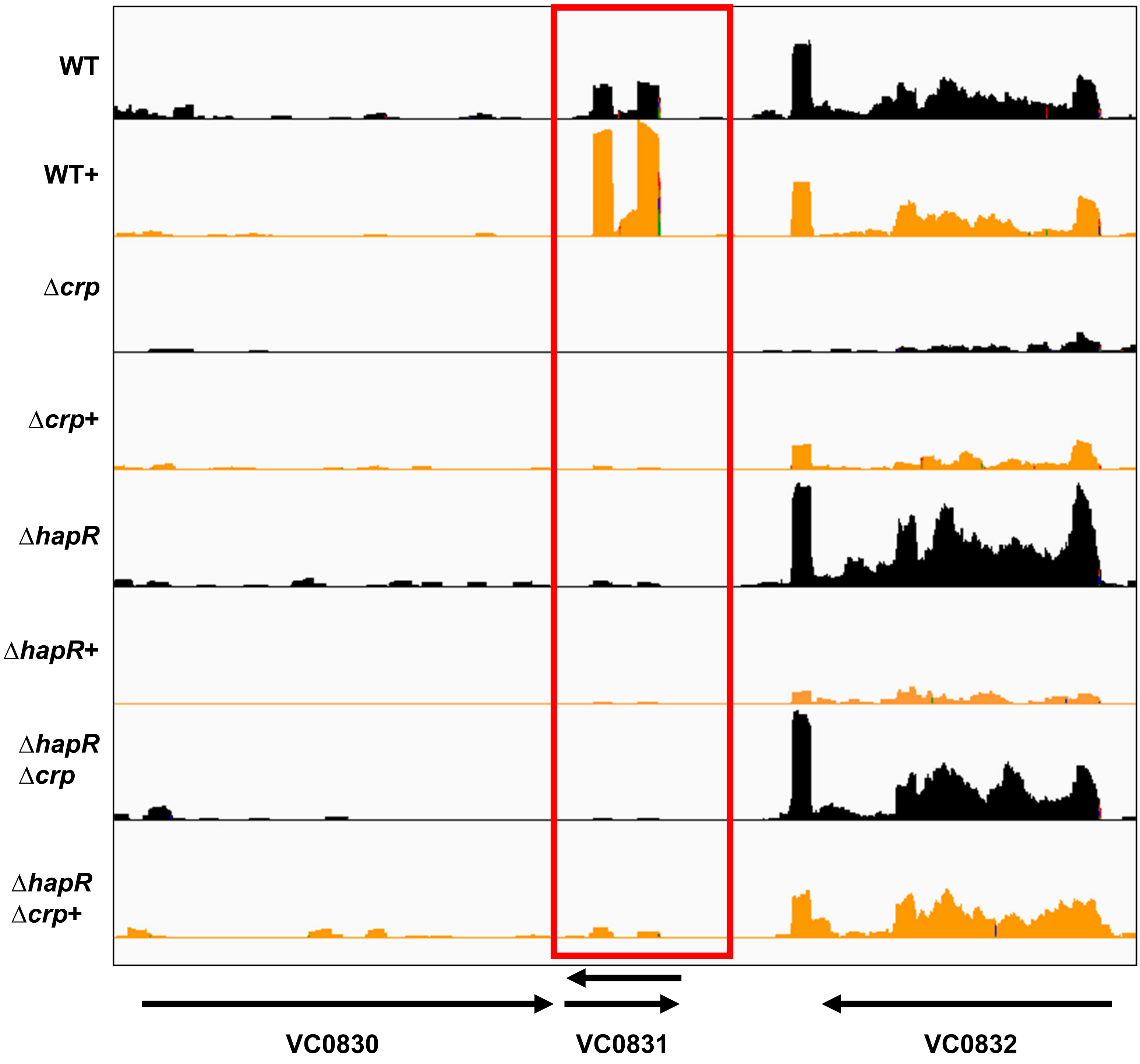

Figure S5: *qrrX* is regulated by nitrate but does not follow the pattern expected of a nitrate-responsive biofilm inhibitor. Normalized RNAseq data showing expression of *qrrX*, which is located between VC0830 and VC0832 and highlighted by the red box. Biological triplicates were performed. A representative trace is shown here.

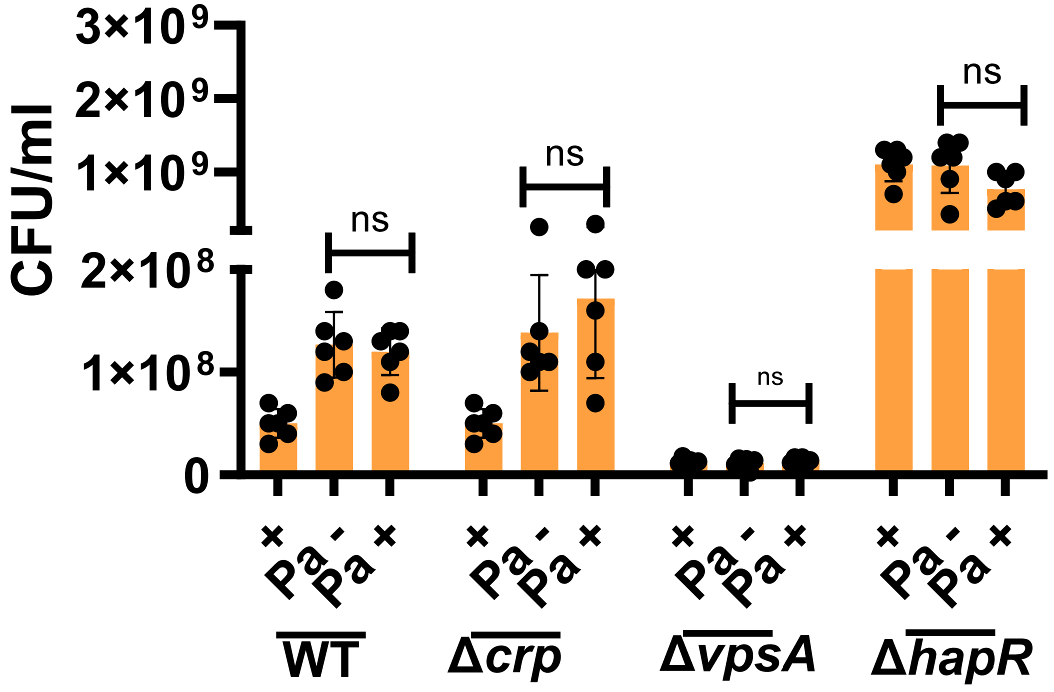

Figure S6: The biofilm formed when *Paracoccus aminovorans* and *V. cholerae* are co-cultured in the presence of nitrate consists principally of *V. cholerae* cells. Quantification of total and streptomycin-resistant (Sm) colony forming units (CFU/ml) in biofilms formed by WT *V. cholerae* and the indicated mutants, which are all streptomycin-resistant, alone or in co-culture with streptomycin-sensitive *P. aminovorans* (Pa). Cells were cultured statically for 48 hours in medium supplemented with 5 mM nitrate alone (-) or with added streptomycin (+). Biofilm mass was quantified by dispersal in phosphate buffered saline, plating of serial dilutions, and enumeration of CFU. The mean of six biological replicates is shown. Error bars reflect the standard deviation. Significance was calculated using a student's t test. ns not significant.
